# Role of Microbes in Alleviating Crop Drought Stress: A Review

**DOI:** 10.3390/plants13030384

**Published:** 2024-01-27

**Authors:** Zechen Gu, Chengji Hu, Yuxin Gan, Jinyan Zhou, Guangli Tian, Limin Gao

**Affiliations:** 1 Engineering and Technical Center for Modern Horticulture, Jiangsu Vocational College of Agriculture and Forestry, Jurong 212400, China; guzechen@jsafc.edu.cn; 2 Department of Agronomy and Horticulture, Jiangsu Vocational College of Agriculture and Forestry, Jurong 212400, China; 13814149982@163.com (C.H.); 19515686323@163.com (Y.G.); 23240962@163.com (J.Z.); gltian19@163.com (G.T.); 3 Nanjing Institute of Agricultural Sciences in Jiangsu Hilly Area, Nanjing 210014, China

**Keywords:** drought stress, crop growth, yield, microbial communities, root exudate, morpho-physiological alteration, biochemical process, molecular response

## Abstract

Drought stress is an annual global phenomenon that has devastating effects on crop production, so numerous studies have been conducted to improve crop drought resistance. Plant-associated microbiota play a crucial role in crop health and growth; however, we have a limited understanding of the key processes involved in microbiome-induced crop adaptation to drought stress. In this review, we summarize the adverse effects of drought stress on crop growth in terms of germination, photosynthesis, nutrient uptake, biomass, and yield, with a focus on the response of soil microbial communities to drought stress and plant-microbe interactions under drought stress. Moreover, we review the morpho-physiological, biochemical, and molecular mechanisms underlying the mitigation effect of microbes on crop drought stress. Finally, we highlight future research directions, including the characterization of specific rhizosphere microbiome species with corresponding root exudates and the efficiency of rhizobacteria inoculants under drought conditions. Such research will advance our understanding of the complex interactions between crops and microbes and improve crop resistance to drought stress through the application of beneficial drought-adaptive microbes.

## 1. Introduction

Drought is currently one of the most serious global issues threatening crop growth and productivity, which occurs every year and has devastating effects on crop yields. A meta-analysis found that crops subjected to drought stress suffered a 48% reduction in yield and concluded that global crop losses from drought stress likely exceeded those from all other abiotic stresses combined [1,2]. Furthermore, a continuous increase in global drought severity is predicted for the future, generating serious plant growth problems for over 50% of arable land by 2050 [3,4].

Water shortages have attracted substantial concern and stimulated growing research on plant drought resistance. Under drought stress, plant morphological, physiological, and biochemical processes are adversely affected by inhibited photosynthesis, cell elongation, cell division, and cell turgor pressure. Plants generally adopt three strategies to overcome drought stress: (1) escape, for example, by accelerating or slowing the switch from vegetative to reproductive growth for avoiding complete abortion under the condition of severe drought stress; (2) avoidance via modulation of the root system architecture, adaptation of leaf traits, and the alteration of photosynthesis; and (3) tolerance, including osmotic adjustment, activation of antioxidant defense systems, and responses to phytohormones and chlorophyll content [4,5].

Plant-associated microbiota play an important role in crop growth and have the potential to alleviate the adverse impacts of drought stress on crops in a sustainable manner [6]. In recent years, rapid theoretical and technological advances in soil microbiology have elucidated the effects of the soil microbiome on plant drought resistance. However, our understanding of the key processes underlying microbiome-induced crop adaptations under drought stress remains limited because of the complexities inherent in the rhizosphere and plant cellular systems. Therefore, in this review, we highlight recent research advances related to the following aspects: (1) the adverse impacts of drought stress on crop growth, such as germination, photosynthesis, nutrient uptake, biomass, and yield; (2) the response of soil microbial communities to drought stress; (3) plant–microbe interactions under drought stress; and (4) the morpho-physiological, biochemical, and molecular mechanisms underlying the microbial mitigation of drought stress. Finally, we discuss future research directions to advance our understanding of the complex interactions between soil, crops, and microbes, which is essential for developing microbial biofertilizers as a biologically potent strategy for microbe-mediated drought tolerance.

## 2. Adverse Impacts of Drought Stress on Crop Growth

**Figure 1:**
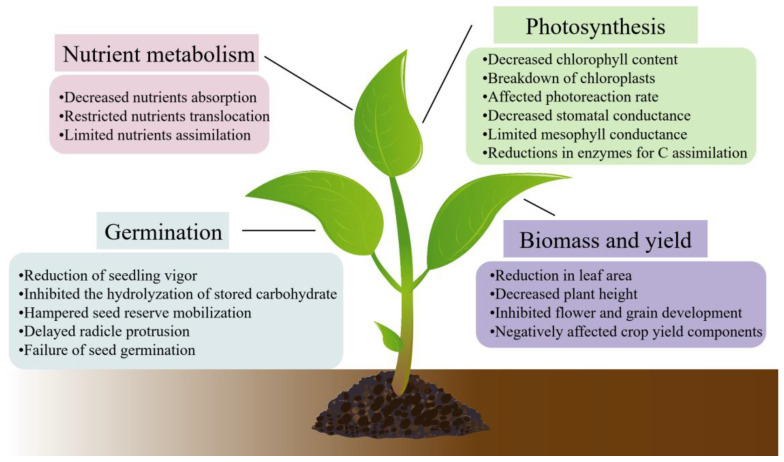
Effect of drought stress on crop growth.

Under conditions of water stress, crops typically exhibit suboptimal growth, reduced leaf area, increased root-to-shoot ratios, and premature senescence. Prolonged drought adversely affects numerous plant cellular functions, including nutrient uptake and assimilation, photosynthesis, protein synthesis, and the accumulation of reactive oxygen species (ROS). An overview of the various detrimental morphological and physiological effects of drought stress on crop growth is depicted in Figure 1.

### 2.1. Germination

Successful seed germination is crucial for seedling establishment and subsequent growth in the field. Seed germination and early seedling growth are the most sensitive stages to water scarcity in many crops, with drought stress negatively impacting parameters such as seed germination index, energy allocation, and seed vigor index across various crop species. The germination of non-dormant seeds unfolds in three distinct phases when a desiccated seed is immersed in water. Phase I of the germination process involves imbibition and water absorption. However, under drought stress, the water potential gradient between the inside and outside of the seed diminishes, impeding water infiltration into the seed coat and subsequent water absorption, ultimately resulting in reduced seedling vigor and inhibited germination [7]. In Phase II, which is known as the lag phase, water uptake ceases, and reserve mobilization commences. Drought stress decreases the activity of hydrolytic enzymes such as amylase, which catalyzes the breakdown of starch into simpler sugars, thereby prolonging the time required for carbohydrate hydrolysis and resulting in failed seed germination [8,9,10,11]. Plant hormones, such as gibberellic acid (GA) and abscisic acid (ABA), play pivotal roles in seed germination; GA activates hydrolytic enzymes, while ABA exerts an opposing influence. Water deficiency triggers an increase in ABA levels but a decrease in GA levels, thus failing to stimulate the synthesis of hydrolytic enzymes and impeding reserve mobilization [12,13,14]. Beyond hydrolytic enzyme activity, the activities of other enzymes such as α-amylase, β-amylase, and proteases are also adversely affected by water deficiency, further hindering seed reserve mobilization [15,16]. Phase III is marked by radicle emergence from the seed coat [8]. In the case of common beans, radicle emergence remains unaffected when drought stress is solely applied during Phase II, whereas drought stress applied after Phase II delays radicle emergence [17].

Drought stress results in the excessive generation of ROS, including superoxide anions (·O_2_^−^), hydrogen peroxide (H_2_O_2_), and hydroxyl radicals (OH·), the accumulation of which leads to DNA or RNA damage, enzyme inactivation, ionic imbalances, and protein denaturation, and ultimately manifests as various physiological and biochemical disruptions in plants [18,19]. An efficient antioxidative defense system in plants regulates the excessive production of ROS through the action of several antioxidant enzymes, including superoxide dismutase, catalase, and peroxidase. However, under drought stress, ROS production surpasses the capacity of the antioxidant defense system owing to impaired enzymatic activity, rendering it incapable of scavenging the elevated ROS levels [20,21]. Consequently, impaired metabolic activity, reduced respiration, and diminished ATP production culminate in the failure of seed germination [22].

### 2.2. Nutrient Metabolism

Nutrient availability and equilibrium are indispensable for optimizing crop growth and yields. Typically, the negative effects of drought stress on crop growth arise from disruptions in nutrient absorption and utilization. Reduced nutrient absorption under drought stress is partially attributed to a decreased nutrient supply via mineralization and an inhibited nutrient movement through diffusion and mass flow, resulting in stunted crop growth, decreased leaf areas, reduced transpiration rates, and a diminished nutrient uptake [23,24,25]. Roots play a vital role in nutrient uptake in crops; under soil water deficits, plants dynamically adapt by modifying their root system architecture through adjustments in root growth patterns [26,27]. While some plants exhibit robust root growth in the early stages of drought stress to access deep soil water, severe soil water deficits can reduce root elongation, branching, and root tip development [5]. Thus, restricted root growth under severe drought stress offers another explanation for reduced nutrient absorption [7]. Additionally, alterations in hormonal concentrations, such as an increase in jasmonic acid, can also suppress nutrient uptake [28].

Limited nutrient translocation under drought stress results from reduced transpiration rates, inhibited active transportation, and decreased membrane permeability [29,30,31]. The expression of various nutrient transporters and channels under drought stress, including nitrate transporter 1.5 (NRT1.5), the ammonium transporter (AMT) family, the phosphate transporter/phosphate transporter family (PHT/PHO/PT) family, and stelar K^+^ outward rectifying (SKOR), represents a critical parameter reflecting the degree of drought tolerance in crops [32]. Furthermore, drought stress compromises the nutrient-assimilation-related enzymes activities [33,34], and the reduced energy availability for nutrient assimilation, such as NO_3_^−^, NH_4_^+^, PO_4_^3−^, and SO_4_^2−^, also leads to a suboptimal nutrient status under drought stress [35].

### 2.3. Photosynthesis

Chlorophyll, as the primary photosynthetic pigment, plays a pivotal role in determining photosynthetic capacity and consequently crop yield. A decreased chlorophyll content is commonly associated with drought exposure in crops such as wheat [36], quinoa [37], soybean [38], and canola [39]. Genotypes with a higher chlorophyll content consistently exhibit superior photosynthetic abilities and, consequently, higher crop yields [40]. A reduced chlorophyll content is partly attributable to the reduced accessibility to essential nutrients such as N, Fe, and Mg, which are critical for chlorophyll synthesis [41]. The accumulation of ROS under drought stress triggers the breakdown of chloroplasts, resulting in the breakdown of chlorophyll precursors and increased chlorophyllase activity [42]. Furthermore, an imbalance between light capture and utilization, resulting from changes in the chlorophyll-to-carotenoid ratio, adversely affects photoreaction rates [18,43]. Varied responses of carotenoids to water deficiency have been reported in the literature, with some studies noting a decreased carotenoid content in plants such as wheat, sorghum, and sunflower [44,45,46,47,48,49], with others indicating enhanced carotenoid contents [38,50,51]. These divergent findings likely arise from differences in species, drought stress duration, and intensity. Carotenoid contents are likely to reduce and increase under moderate and severe drought stress, respectively, where they play a crucial role in the plant antioxidant defense system by mitigating oxidative damages caused by drought stress.

Water stress affects both the light and dark reactions, consequently reducing the accumulation of photosynthetic products. The reduced stomatal conductance under water stress, driven by an elevated ABA content in guard cells, has been extensively studied [52,53,54,55,56]. ABA-mediated stomatal closure represents the primary response of plants to drought stress, serving as a mechanism to reduce water loss through transpiration, thereby preserving hydraulic function but limiting CO_2_ intake, ultimately leading to decreased photosynthesis rates [4,56,57,58,59].

Crop photosynthesis is also constrained by mesophyll conductance. Non-stomatal limitations of photosynthesis (mesophyll conductance or Rubisco carboxylation capability) are substantially more pronounced under water stress conditions relative to stomatal limitations [60]. Mesophyll conductance primarily depends on leaf anatomical characteristics such as leaf and cell wall thickness, and chloroplast morphology [61]. A reduced mesophyll conductance and, consequently, lower photosynthesis rates largely depend on crop species, stress duration, and intensity [62]. Roig-Oliver et al. [63] concluded that the variations in cell wall thickness significantly correlate with mesophyll conductance under both short- and long-term water deficit stress, whereas other research suggests that a reduced chloroplast surface area exposed to leaf intercellular air spaces per leaf area (*S*_c_/*S*), influenced by chloroplast size, number, and arrangement, contributes to a diminished mesophyll conductance under drought stress in rice, wheat, and cotton [64,65,66,67,68]. Furthermore, reduced aquaporin and carbonic anhydrase activities play a role in reduced CO_2_ diffusion within the mesophyll under drought stress [67,69,70]. Insufficient chloroplastic CO_2_ concentrations during water scarcity lead to the accumulation of reduced photosynthetic electron transport components, potentially influencing molecular oxygen generation and resulting in ROS production [71]. ROS overaccumulation induces the oxidation of chloroplast proteins, DNA, and lipids [7], along with the reduction in enzymes essential for carbon assimilation and utilization [72,73]. For example, the activity and abundance of ribulose-1,5-bisphosphate carboxylase/oxygenase (Rubisco) dramatically decline under water deficiency, negatively impacting photosynthesis [49].

### 2.4. Biomass and Yield

Drought represents a significant factor contributing to reduced yields and food shortages [74]. Insufficient water during both the early vegetative and reproductive stages substantially hampers crop biomass and yield. Limited water and nutrient uptake and reduced photosynthetic rates impede cell expansion, resulting in a diminished leaf area and restricted internode elongation and plant height [43]. Furthermore, during stem elongation, vegetative growth predominantly determines the final yield, and reduced carbon assimilation leads to impaired flower and grain development [75]. Drought exerts a more pronounced detrimental effect during the reproductive phase, wherein a water deficit not only hinders flower initiation, and ovary and embryo development, but also impacts pollen development, flowering, fertilization, and grain development [76]. Key crop yield components are significantly and negatively affected by water scarcity in crops, such as rice, wheat, cotton, potato, maize, soybean, and sugar beet [18,77,78,79,80,81]. By a meta-analysis, Zhang et al. [82] reported that wheat and rice yields declined by 27.5% and 25.4% under drought stress, respectively.

## 3. Plant Microbiome Response to Drought Stress

The microbiome, which is considered as the second genome of the plant, is critical for plant growth, especially in unfavorable environments. Drought-triggered changes in crop fungal communities have been studied extensively. For example, large shifts in root-associated fungal communities have been reported under drought conditions in castor bean, with *Fusarium*, Chytridiomycota, Ascomycota, and Basidiomycota dominating the rhizosphere and bulk soil [83]. Compared with bulk soil and the rhizosphere, the grapevine root endosphere compartment shows the greatest divergence in fungal composition under severe drought stress, with the Chao1 and Shannon diversity of the fungal communities in the root being significantly lower than those in the absence of water deficiency. A significant enrichment in arbuscular mycorrhizal fungus (AMF) *Funneliformis* was also identified within the roots during drought, which is predominantly attributed to decreased P availability in drought soil [84,85]. Moreover, specific fungal species have been identified in wheat roots under drought stress; for example, *Trichoderma longibrachiatum* and *T. velutinum* are only identified under drought stress, whereas *Zopfiella* sp., *M. hedericola*, *A. verrucaria*, *G. radicicola*, and *A. salicis* are observed specifically in irrigated plant groups [86]. Changes in the composition of root-associated fungal communities and increased fungal biodiversity in rice plants have also been reported, with the majority of identified OTUs belonging to the Pezizomycotina subphylum [87]. The response of phyllosphere fungal communities to water stress has been studied in tomatoes, whereby drought stress increases the evenness and therefore the Shannon diversity index of fungal communities, and the relative abundance of dominant fungal taxa is also reduced by water deficiency [88]. These results verify that drought stress restricts compartment-specific fungal communities. Using sorghum as a research system, Gao et al. [89] concluded that, under drought stress, the stochastic force was the major force affecting fungal community assembly in leaves and roots. Moreover, they demonstrated that the host compartment was the strongest driving factor influencing fungal assembly, followed by the timing of plant development, and finally the plant genotype. Azarbad et al. [90] concluded that the effect of water stress on fungal diversity depends on the genotype and soil history, as shown by increased fungal diversity in the rhizosphere of wheat growing in soils with a history of water stress; in contrast, no significant changes in rhizosphere fungal α-diversity were found in soils, which never suffers from water stress. 

Wheat grown in soils under water stress harbors more fungi and fewer bacteria [91]. Furthermore, bacterial biodiversity is decreased in the soil but increased in the root endosphere [92]. In rice plants, Si et al. [92] found that drought exerts a negligible effect on the alpha diversity of rhizosphere bacterial communities, but substantially enriches Actinobacteria and decreases Firmicutes [93]. Similarly, Santos-Medellin et al. [94] concluded that drought alters bacterial composition in the endosphere and rhizosphere compartments of rice, characterized by an enrichment of *Actinobacteria* and *Chloroflexi* but a depletion of *Acidobacteria* and *Deltaproteobacteria*. Specifically, the relative abundances of Actinobacteria and Acidobacteria were increased in peanut seedlings and podding stages under drought stress, whereas the relative abundances of Cyanobacteria and Gemmatimonadetes were increased in the flowering stage [95]. Furthermore, in millet plants, Simmons et al. [96] proved that drought intensity is correlated with the enrichment level of *Actinobacteria*. In conclusion, Actinobacteria enrichment within drought-stressed root microbiomes is strongly conserved among evolutionarily diverse plant species. Moreover, decreases in the phyla Proteobacteria and Verrucomicrobia, as well as increases in the ratio of Gram-positive to Gram-negative bacteria, are also frequently observed under drought conditions [93,97,98]. Drought significantly restructures the sorghum root microbiome composition and functionality during its early stage, which coincides with an increased abundance and activity of monodermal bacteria [99]. The taxonomic patterns in the phyllosphere are totally different from those detected in soil and rhizosphere studies. In the tomato phyllosphere, water stress reduces both leaf bacterial richness and the Shannon index, while simultaneously enriching several *Proteobacteria* and *Bacteroidetes* families and depleting several *Actinobacteria* families [88].

## 4. Plant–Microbe Interaction under Drought Stress

Changes in crop morphology and metabolism under drought conditions govern the responses of microbial communities [100]. Drought stress induces an increase in root depth and density and therefore an increase in the root/shoot ratio, which affects the composition and distribution of microbiomes and vice versa [19]. Considering the inhibition of photosynthetic activity due to stomatal closure, a previous study found that the abundance of 163 metabolites changed significantly during water stress in soybean roots [101]. Plants regulate their rhizosphere composition to promote the growth of microorganisms and improve plant adaptability in a given ecosystem, with root exudates being the ultimate designers of the rhizosphere microbiome [102,103]. In general, root exudates, including soluble sugars, amino acids, and organic acids, increase with drought stress intensity [104]. Altered carbon exudation through rhizodeposition disrupts microbial processes and shapes root-associated microbial communities [105,106]. Preece and Peñuelas [105] concluded that the effects on rhizodeposition are strongly species-dependent; therefore, the impacts on soil communities also vary. Among the various classes of compounds, the glycolysis intermediate glycerol-3-phosphate, which is a precursor for peptidoglycan biosynthesis and cell wall formation in bacteria [107], accumulates significantly under water deficit stress in sorghum [99]. Strong associations between rhizosphere bacterial communities and root exudates have also been confirmed in rice plants subjected to drought stress. Specifically, *Streptomyces* exhibits a positive correlation with ABA and a negative correlation with jasmonic acid, whereas some members of Actinobacteria (*Conexibacter*, *Gaiella*, *Marmoricola*, and *Nocardioides*) are positively associated with L-threonine, L-threonine, L-valine, and L-tryptophan [108].

Moreover, the dominant role of the host plant genotype in plant–microbe interactions under drought stress is associated with the root exudates. The host root zone, where enriched compounds are secreted by both plants and microorganisms, plays a key role in maintaining plant–microbe interactions [109]. For example, the abundance of OTUs and more saprotrophs enriched in the rhizoplane and rhizosphere of drought-tolerant species is far greater than that in the sensitive species in sugarcane, which contributes to enhanced drought resistance by altering the ABA pathways and, therefore, the root exudates [110]. In a drought-tolerant rice plant genotype, an increase in the relative abundance of *Bacillus* was found to depend on upregulated amino acid exudation [108].

## 5. Mechanisms Underlying the Mitigation Effect of Microbes on Crop Drought Stress

**Figure 2:**
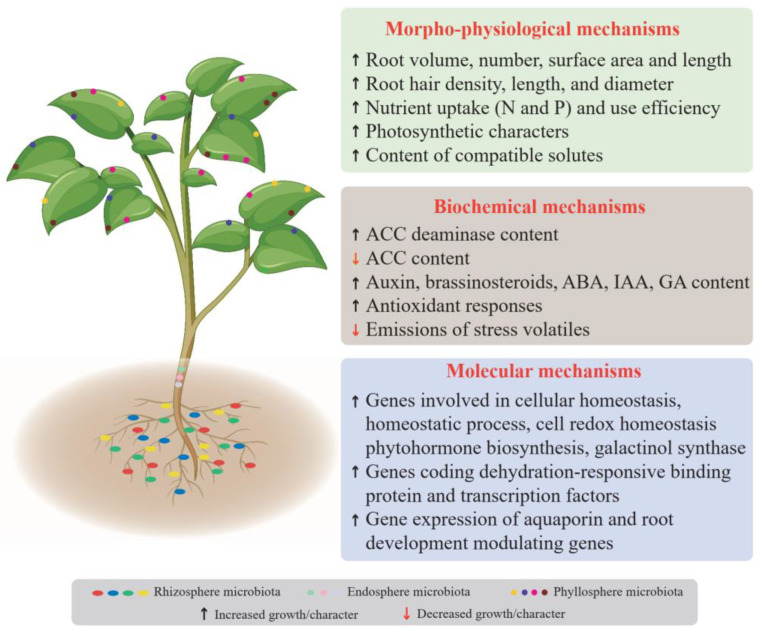
Mechanisms underlying the effect of microbes on crop drought resistance.

Under water stress conditions, crop systems actively maintain their physiological water balance either by increasing water uptake or reducing water loss through closing the stomata. Moreover, osmotic regulation is also one of the key measures for plants to cope with drought [4]. Activated stress response pathways regulated by microbes are closely associated with changes in crop morpho-physiology, biochemical processes, and molecular responses (Table 1, Figure 2).

### 5.1. Morpho-Physiological Mechanisms

Longer roots are profitable for plants to obtain nutrients and water from more soil, and roots respond to changes in soil water content at both the cellular scale and with the entire root system architecture [4]. Under severe drought stress, inoculation with osmotolerant endophytic bacteria increases the root volume, area, and length in pearl millet [112]. Mycorrhizal fungal colonization also significantly increases the volume, number, and length of lateral roots, as well as the root hair density, length, and diameter under drought stress conditions in tea plants [111]. *G. diazotrophicus* ameliorates root growth under water-restricted conditions by increasing the root length and number of lateral roots in rice plants [116]. According to a meta-analysis, AM-inoculated plants have a 49% greater root dry weight than non-mycorrhizal plants under drought stress, which is the result of increased root length (37%), root surface (31%), and root volume (65%) [135]. Moreover, the coleoptiles of wheat seedlings inoculated with *Azospirillum* show wider xylem vessels and a less negative water potential under water stress, which explains their improved water status after inoculation [132]. 

Physiologically, plant-growth-promoting bacteria (PGPR) or AMF inoculation significantly ameliorates the negative effects of drought by increasing nutrient (N and P) uptake, use efficiency, and photosynthetic characteristics such as chlorophyll synthesis, the potential quantum efficiency of PSII (Fv/Fm), non-photochemical efficiency, photochemical efficiency, actual quantum yield, and photosynthesis rate in maize [113,117], chickpea [118], soybean [121], sorghum [123], and grapevines [114,135]. An increased content of compatible solutes, such as proline, sugars, and free amino acids, is also associated with preservation of the relative water content and, therefore, drought stress resistance [113,119,120,136].

### 5.2. Biochemical Mechanisms

An increase in the synthesis of ethylene from its immediate precursor 1-aminocyclopropane-1-carboxylate (ACC) [137], secreted by plants as a root exudate, has been observed in almost all plants growing under stress conditions. Inoculation with *V. paradoxus* 5C-2 improves growth, yield, and water-use efficiency during drought by amplifying the xylem ABA concentration and attenuating the xylem ACC concentration [43,134]. The combined effect of the three ACC-deaminase-producing rhizobacteria in *Vigna mungo* L. and *Pisum sativum* L results in decreased ACC accumulation and downregulated ACC-oxidase gene expression [122]. *Bacillus subtilis* provides tolerance to drought stress in wheat by increasing the indole-3-acetic acid content and counteracting the increased ACC content [124]. The application of endophytes or AMF in the alleviation of drought stress, either by producing ACC deaminase or increasing the content of hormones, such as auxin, brassinosteroids, ABA, indole-3-acetic acid, and GA, has also been clarified in soybean [115], mung bean [126], *Capsicum annuum* [125], pearl millet [112], *Alhagi sparsifolia* [138], tea [111], and wheat [133]. In the presence of *Bacillus safensis*, enhanced antioxidant responses occur because of the elevated activities of enzymes, including catalase, peroxidase, ascorbate peroxidase, superoxide dismutase, and glutathione reductase in wheat plants, which contributes to their outstanding resistance to water stress [130]. Activated antioxidant enzyme activity after AMF inoculation under drought stress has also been observed in *Alhagi sparsifolia* [138], wheat [129], and mung bean [131]. Moreover, reduced emissions of stress volatiles, including benzaldehyde, β-pinene, and geranyl acetone, are likely candidates responsible for drought stress mitigation in the early stress stages [129].

### 5.3. Molecular Mechanisms

After 1 h of drought stress induction, 194 genes with modified expression in *B. phytofirmans* PsJN colonizing non-stressed and drought-stressed potato plants were identified. These genes were upregulated in the transcriptome mostly in the following functional categories: cellular homeostasis, homeostatic processes, and cell redox homeostasis [128]. Moreover, the positive impact of PGPR inoculation on drought resistance is associated with the modulated expression of a regulatory component (CTR1) of the ethylene signaling pathway and the DREB2 transcription factor [124,131]. Similarly, the dehydration-responsive binding protein *Gmdreb1a* and the galactinol synthase *Gmgols* were strongly expressed in response to drought stress in the presence of *Bacillus cereus* in soybeans [121]. Except for *DREB2A*, the expression levels of genes involved in phytohormone biosynthesis (*SbNCED*, *SbGA20oX*, and *SbYUC*) and those coding for drought-responsive transcription factors (*SbAP2* and *SbSNAC1*) are significantly higher in endophyte-inoculated plants than in uninoculated pearl millet plants subjected to severe drought stress [112]. Furthermore, the differential expression of genes involved in ROS scavenging (*CAT*, *APX*, *GST*), ethylene biosynthesis (*ACO* and *ACS*), salicylic acid (*PR1*), and jasmonate (*MYC2*) signaling has also been observed under drought conditions in PGPR-inoculated plants, in contrast to uninoculated control plants [131]. Colonization with *T. harzianum* can alter the expression of aquaporin and dehydrin genes in rice genotypes under drought stress, thereby contributing to the advancement in sustained crop productivity under drought stress [127]. Additionally, reverse transcription PCR analysis of rice roots revealed significant differences in the expression of root-development-modulating genes during the water restriction period, with the relative gene expression increasing 10–50-fold compared to uninoculated field-grown plants [116].

## 6. Future Perspectives

In this review, we have addressed the primary impacts of drought stress on crop growth and the intricate interplay between crops and microorganisms in drought-prone environments. We have systematically elucidated the underlying mechanisms governing how microorganisms can ameliorate the adverse effects of drought stress on crops. The body of evidence presented herein unequivocally highlights the profound influence of drought stress on crop responses, particularly in shaping the composition of root microbial communities, with root exudates emerging as pivotal contributors to this dynamic process. Nevertheless, a substantial research gap exists in the characterization of root exudates across diverse crop species. The complex milieu of hundreds of chemically complex primary and secondary metabolites exuded from roots, compounded by simultaneous releases by microbes, renders the identification of specific root exudates a formidable task.

Given the pivotal role of root exudates in modulating root microbial communities, the application of exudates holds significant promise for mitigating the adverse effects of drought stress on crop growth. Hence, sustained efforts are required to characterize and quantify root exudates under drought stress conditions.

Furthermore, microbial inoculants are the best possible candidates for promoting healthier and more sustainable food production while bolstering environmental friendliness and agricultural sustainability. A deeper exploration of distinct rhizobacterial microbes could enhance our understanding of their interconnectedness and their potential to fortify crops against drought stress. However, many questions remain unanswered. First, specific rhizosphere microbiome species designed using the corresponding root exudates are difficult to discriminate. Second, research into the efficiency of rhizobacteria inoculants under drought conditions is limited. Nevertheless, the application of beneficial drought-adaptive microbes to crops could be a sustainable solution enabling plants to withstand stress conditions, paving the way for improved economic yields, soil health, and fertility. Thus, considerable future research is required to identify optimal rhizobacterial strains and potential field candidates, as well as to develop more effective PGPR strains with longer shelf-life to ensure sustainable crop production in arid areas. 

## Figures and Tables

**Table 1:** Effects of microbes on crop drought resistance and related mechanisms.

Crops	Microbes	Related Mechanisms				References	DOI
		Morphological	Physiological	Biochemical	Molecular		
Tea	*Claroideoglomus etunicatum*					Liu et al. [111]	https://doi.org/10.1007/s10725-023-00972-8
Pearl millet	*Shewanella putrefaciens*, *Cronobacter dublinensis*					Manjunatha et al. [112]	https://doi.org/10.1111/ppl.13676
Maize	*Sphingobacterium changzhouense*					Hagaggi and Abdul-Raouf [113]	https://doi.org/10.1007/s11274-022-03441-y
Grape	AMF					Ye et al. [114]	https://doi.org/10.3390/agronomy12071563
Soybean	*Bacillus cereus*, *Pseudomonas otitidis*, *Pseudomonas* sp.					Dubey et al. [115]	https://doi.org/10.3390/ijerph18030931
Rice	*Gluconacetobacter diazotrophicus*					Silva et al. [116]	https://doi.org/10.3390/ijms21010333
Maize	PGPR *Cupriavidus necator* (B1), *Pseudomonas fluorescens* S3X					Pereira et al. [117]	https://doi.org/10.1016/j.heliyon.2020.e05106
Chickpea	AMF					Hashem et al. [118]	https://doi.org/10.1016/j.sjbs.2018.11.005
Maize	AMF					Begum et al. [119]	https://doi.org/10.3390/plants8120579
Wheat	*Alternaria alternata*					Qiang et al. [120]	https://doi.org/10.1007/s11104-019-04028-7
Soybean	*Bacillus subtilis*, *Bacillus thuringiensis*, *Bacillus cereus*					Martins et al. [121]	https://doi.org/10.1007/s11356-018-1610-5
*Vigna mungo* L.	*Ochrobactrum pseudogrignonense* RJ12, *Pseudomonas* sp. RJ15 *and Bacillus subtilis* RJ46					Saikia et al. [122]	https://doi.org/10.1038/s41598-018-25174-5
*Pisum sativum* L.	*Ochrobactrum pseudogrignonense* RJ12, *Pseudomonas* sp. RJ15 *and Bacillus subtilis* RJ46					Saikia et al. [122]	https://doi.org/10.1038/s41598-018-25174-5
Sorghum	*Exophiala pisciphila* GM25					Zhang et al. [123]	https://doi.org/10.17957/IJAB/15.0241
Wheat	*Bacillus subtilis*					Barnawal et al. [124]	https://doi.org/10.1111/ppl.12614
*Capsicum annuum*	*Bulkhorderia cepacian*, *Citrobacter feurendii*, *Serratia marcescens*					Maxton et al. [125]	https://doi.org/10.1080/01904167.2017.1392574
Mung bean	*Pseudomonas simiae*					Kumari et al. [126]	https://doi.org/10.1007/s11274-015-1974-3
Rice	*Trichoderma harzianum*					Pandey et al. [127]	https://doi.org/10.1007/s00425-016-2482-x
Potato	*Burkholderia phytofirmans*					Sheibani-Tezerji et al. [128]	https://doi.org/10.1128/mBio.00621-15
Wheat	*Bacillus thuringiensis* AZP2, *Paenibacillus polymyxa* B					Timmusk et al. [129]	https://doi.org/10.1371/journal.pone.0096086
Wheat	*Bacillus safensis*, *Ochrobactrum pseudogregnonense*					Chakraborty et al. [130]	https://doi.org/10.1007/s11274-012-1234-8
Mung bean	*Pseudomonas aeruginosa* GGRJ21					Sarma and Saikia [131]	https://doi.org/10.1007/s11104-013-1981-9
Wheat	*Azospirillum brasilense* Sp245					Pereyra et al. [132]	https://doi.org/10.1016/j.apsoil.2011.11.007
Wheat	*Azospirillum lipoferum*					Arzanesh et al. [133]	https://doi.org/10.1007/s11274-010-0444-1
Pea	*Variovorax paradoxus* 5C-2					Belimov et al. [134]	https://doi.org/10.1111/j.1469-8137.2008.02657.x

Note: Green, blue, yellow and pink box represented morphological, physiological, biochemical, and molecular mechanisms were involved in the corresponding references, respectively.
